# Proton pump inhibitor use: systematic review of global trends and practices

**DOI:** 10.1007/s00228-023-03534-z

**Published:** 2023-07-07

**Authors:** Lelwala Guruge Thushani Shanika, Andrew Reynolds, Sharon Pattison, Rhiannon Braund

**Affiliations:** 1 New Zealand Pharmacovigilance Centre, University of Otago, 913, Dunedin, 9054 New Zealand; 2 Department of Pharmacy and Pharmaceutical Science, University of Sri Jayewardenepura, Gangodawila, Sri Lanka; 3 Department of Medicine, Dunedin School of Medicine, University of Otago, Dunedin, New Zealand

**Keywords:** Dose, Global use, Long term, Proton pump inhibitors, Systematic review

## Abstract

**Purpose:**

Proton pump inhibitors (PPIs) reduce acid secretion in the stomach and rank as one of the most widely used acid-suppressing medicines globally. While PPIs are safe in the short-term, emerging evidence shows risks associated with long-term use. Current evidence on global PPI use is scarce. This systematic review aims to evaluate global PPI use in the general population.

**Methods:**

Ovid MEDLINE, Embase, and International Pharmaceutical Abstracts were systematically searched from inception to 31 March 2023 to identify observational studies on oral PPI use among individuals aged ≥ 18 years. PPI use was classified by demographics and medication factors (dose, duration, and PPI types). The absolute numbers of PPI users for each subcategory were summed and expressed as a percentage.

**Results:**

The search identified data from 28 million PPI users in 23 countries from 65 articles. This review indicated that nearly one-quarter of adults use a PPI. Of those using PPIs, 63% were less than 65 years. 56% of PPI users were female, and “White” ethnicities accounted for 75% of users. Nearly two-thirds of users were on high doses (≥ defined daily dose (DDD)), 25% of users continued PPIs for > 1 year, and 28% of these continued for > 3 years.

**Conclusion:**

Given the widespread use PPIs and increasing concern regarding long-term use, this review provides a catalyst to support more rational use, particularly with unnecessary prolonged continuation. Clinicians should review PPI prescriptions regularly and deprescribe when there is no appropriate ongoing indication or evidence of benefit to reduce health harm and treatment cost.

**Supplementary Information:**

The online version contains supplementary material available at 10.1007/s00228-023-03534-z.

## Background

Proton pump inhibitors (PPIs) are weakly basic acid-labile pro-medicines that reduce acid secretion in the stomach [1]. PPIs are a class of medicines which include omeprazole, esomeprazole, pantoprazole, lansoprazole, rabeprazole, and dexlansoprazole.

PPIs are the cornerstone in the management of gastric and duodenal ulcers, dyspepsia, gastro-esophageal reflux disease (GERD), Zollinger–Ellison (ZE) syndrome, Helicobacter pylori (H. pylori) eradication, and prevention and treatment of non-steroidal anti-inflammatory (NSAID)-associated ulcers [2, 3]. There is also widespread “off label” use for prophylaxis against gastritis associated with corticosteroids, anticoagulants, chemotherapy, and coronary heart disease [4, 5].

Given the utility of these medications, safety profile, efficacy, and tolerability, their usage globally is significant. In 2020, omeprazole was ranked as the second-highest dispensed item in England, with almost 35 million prescriptions filled and an annual cost of GBP 82 million [6]. In the USA, omeprazole was the eighth most commonly prescribed medication in 2019 with more than 52 million prescriptions [7]. The USA spent $19.99 billion in 2016–2017 on PPIs [8].

Although PPIs are generally considered safe for short-term use, evidence of serious side effects with long-term use is mounting. These potential risks include increased risk of pneumonia, enteric infection, bone fracture, gastrointestinal tract cancers, and reduced absorption of vitamins and minerals [2, 9].

Indications that require long-term PPI use are limited. Apart from some specific acid-related diseases (e.g., ZE syndrome and Barrett’s esophagus (BO)), it is recommended that PPIs should be discontinued 4 to 8 weeks after initiation [2, 9].

Despite the wide prevalence of their use, cost to health care systems, routine “off-label” use, and evidence that these medications are used for longer than recommended; no systematic reviews have been conducted to examine global trends and practices of PPI use.

We have undertaken this review to fill this appreciable gap in the literature and comment on PPI use in the general population, medication factors (dose, frequency, duration, and PPI type), and demographic factors (age, sex, and ethnicity).

## Methods

This systematic review was conducted in line with Cochrane recommendations [10] and reported according to the Preferred Reporting Items for Systematic Reviews and Meta-analyses (PRISMA) 2020 guidelines [11]. The protocol for this review was registered on the International Prospective Register of Systematic Reviews (PROSPERO) (CRD42020167968) [12].

## Study inclusion criteria

Observational studies (cross-sectional, case–control, and cohort studies) that reported population level PPI use were considered eligible.

Studies were excluded if they were not deemed to be representative of the general population. For example, studies of only males OR females or studies who recruited specific subsets of the population (with pre-existing condition or other medication use).

Hospital use of PPIs was excluded as these may be used for stress ulcer prophylaxis or as a short-term symptomatic relief. However, if the hospital setting was used to recruit long-term PPI users or recruited patients with PPI use on admission, these studies were included. Clinical trials, conference abstracts, case-reports or case series, commentaries, editorials, letters, and non-English articles were not eligible.

## Search strategy

Ovid MEDLINE, Embase, and International Pharmaceutical Abstracts (IPA) were systematically searched from inception to 31 March 2023 to identify observational studies on oral PPI use (liquids and capsules) among individuals aged 18 years and older in the general population. The detailed search strategy is provided in Supplementary File 1.

After removing the duplicates, the references were imported into Excel. Two reviewers (L.G.T.S. and R.B.) independently screened titles and abstracts, and then full text, to assess for eligibility. Disagreements were resolved by consensus between the two reviewers or arbitrated by a third reviewer (S.P.). Reference lists of eligible studies for the full-text analysis, including systematic reviews and meta-analyses, were further hand searched manually by two reviewers to identify any additional articles. Where multiple studies reported from the same data source, the study with the longest time period with the largest number of participants and the highest number of utilization variables was included.

## Data extraction

The following information was extracted from all eligible studies following a template from a previous systematic review [13]: (A) article information (first author and year of publication), (B) study characteristics (country, study period, study design, study setting, and source population) and PPI population (i.e., the population from which eligible PPI users were drawn), (C) patient’s characteristics, and (D) medication characteristics and possible indication. If the data were presented in graph form only, the values were extracted using WebPlotDigitizer version 4.2 [14]. For studies presenting sequential years of national sampling, the most recent year was used to retrieve the data.

PPI use was classified by both [1] patient’s demographics (age, sex, and ethnicity) and [2] medication factors (dose and/or frequency, duration of PPI use, and PPI type) when possible.

Participants of each study were categorized as “prevalent PPI users” (i.e., patients who had already been prescribed PPIs before study recruitment and continued the medicine throughout the study period) or “new PPI users” (i.e., PPI naïve; PPI therapy was newly started and had long-term follow-up data) based on the information provided. Each utilization variable is reported stratified by user type (i.e., new users only and total users (prevalent + new users)).

## Extracted variable standardization

The categories for each variable were not pre-defined, and they were developed by the review team to best capture the majority of available data from individual studies.

### Age

Participant age was categorized into three groups (≤ 49, 50–64, and ≥ 65 years old) based on the available data.

### Sex

All studies reported on sex or gender by binary classification only (male or female).

### Ethnicity

Studies described ethnicity or race differently, using terms such as: White, Black, Asian, Caucasian, African American, Hispanic, Asian, and Other. We collapsed these terms into four groups (White, Black/African American, Asian, and Other).

### PPI types

Data were provided on overall PPI and by specific PPI such as “Omeprazole,” “Esomeprazole,” “Pantoprazole,” “Lansoprazole,” and “Rabeprazole.” For less common PPIs such as dexlansoprazole or other PPI combinations, we categorized these as “Other.”

### Dose

Studies described doses differently, using terms such as: higher dose, maintenance dose, lower dose, and on-demand, that is taking PPI intermittently, when experiencing symptoms. We considered this dose categorization.

For the purposes of this review, higher dose was defined as equal to or higher than the defined daily dose (DDD), while lower dose was defined as being less than the DDD. The World Health Organization (WHO) defines the DDD as the “assumed average maintenance dose per day for a drug used for its main indication in adults” [15].

Dose frequency information was clustered into once daily, twice daily, on-demand, and other.

### Duration

We defined duration of PPI use as either “Short-term” (defined as less than one year) or “Long-term” (defined as greater than one year).

To identify whether PPI use practice complied with current treatment guidelines [2, 9], we calculated the number of users who were on prescriptions of three months or less. Long-term users were further stratified into one year to three years and more than three years use.

### Indication

Indication for use was clustered into eight categories based on clinical similarities. These were gastroprotection (GI irritant medicine or treatment side effects), dyspepsia/GERD, gastritis/duodenitis, ulcer/GI bleeding, *H. pylori* infection, BO/ZE syndrome, uncertain/unknown indication, and other.

On request during peer review, a sub-analysis was undertaken for studies reporting on utilization prevalence, incidence, and characteristics of users that were fully generalizable to the country’s population.

## Statistical analysis

The utilization variables were descriptively assessed using percentages. All analyses were performed using Microsoft Excel (2019).

The absolute numbers of PPI users for each subcategory within a variable were summed up, and the percentages were computed using the total number of individuals for that variable as the denominator.

E.g.,

Total PPI users for age variable = a.

Number of PPI users younger than 40 years = b.

Percentage of PPI users younger than 40 years = (b/a) × 100.

## Results

### Description of included studies

**Fig. 1:**
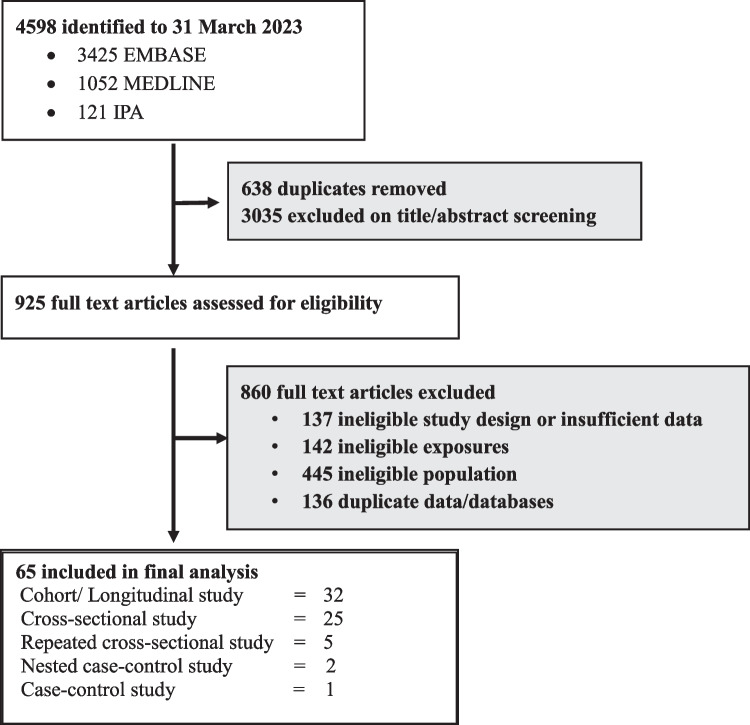
Flowchart of eligible study identification

The process for identifying the included studies is shown in Fig. 1. Online searches identified 4598 records, of which 638 were duplicates. The remaining 3960 articles were screened by titles and abstracts, with 925 articles then screened as full text. The 65 articles identified as eligible to our research question are described in Supplementary Table 1.

**Fig. 2:**
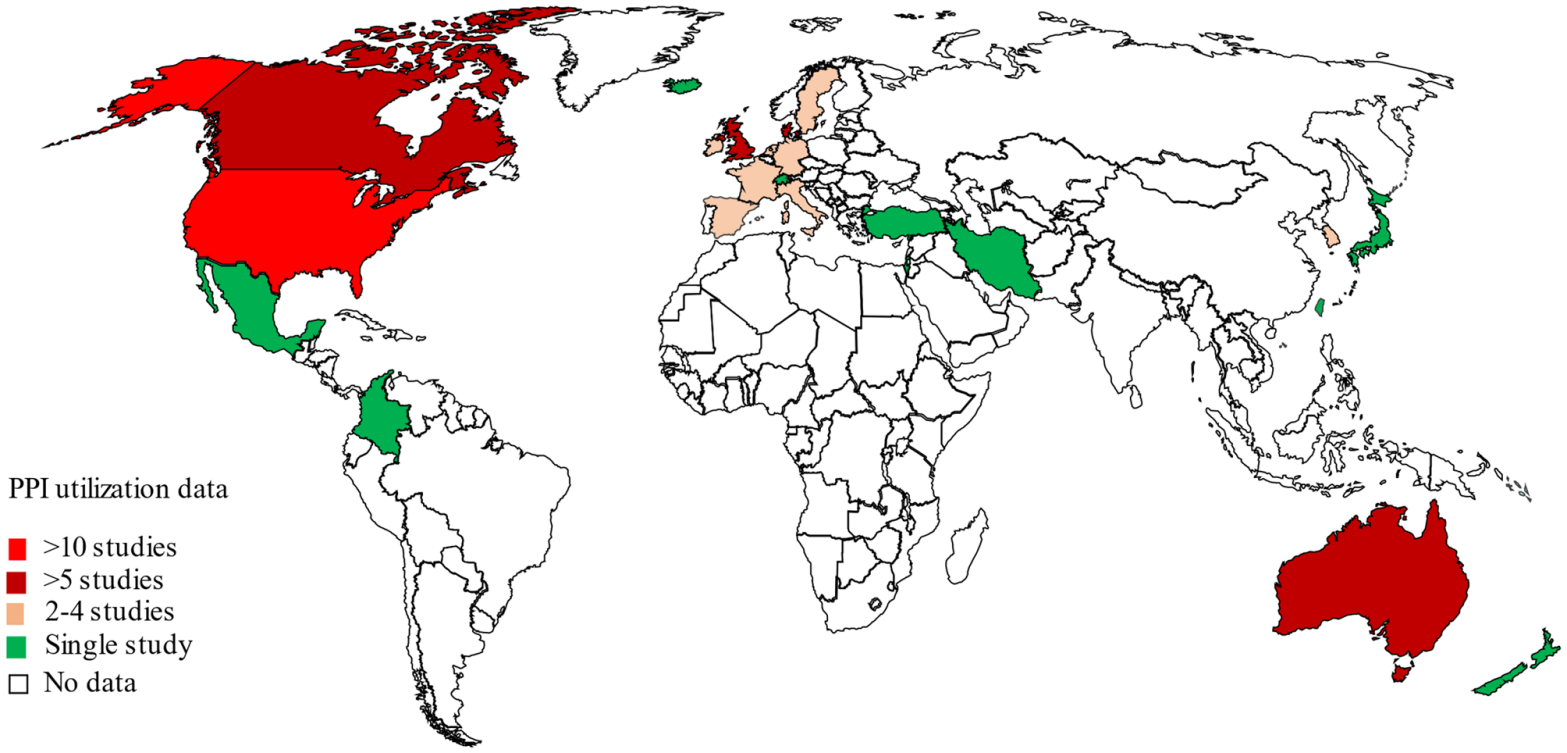
Global regions where PPI utilisation data were available

Eligible studies were carried out between 1988 and 2022 and included data on 28 million prevalent and new PPI users (ranging from 32 to 4,388,586 PPI users in each study). The sixty-five [8, 16–79] included studies were from 23 countries, with all the data from developed countries except Colombia [51], Iran [31], and Mexico [66] (Fig. 2).

### PPI utilization in the general population

Out of 65 studies, 28 reported the source population. From these studies, it was estimated that 23.4% of the adult source population in the world were PPI users (18,326,284/78,151,104). Table 1 presents the number of studies eligible for the analysis of each utilization variable.

**Table 1:** Summary of the number of studies included for analysis of each utilization variable

Variable	No. of studies with data for variable	No. of studies eligible for analysis	No. of studies that could not be included and why
Age	31	26	5 (age was reported as prevalence proportion (*N* = 2) and DDD/1000/day^a^ (*N* = 2) instead of per patient; extreme outlier (*N* = 1))^*^
Sex	63	60	3 (sex was reported as prevalence proportion (*N* = 1), DDD/1000/day (*N* = 1), and prescription counts (*N* = 1) and not per patient)
Ethnicity	10	9	1 (ethnicity was reported as DDD/1000/day (*N* = 1) and not per patient)
Medication types	35	31	4 (PPI types were reported as million DDD (*N* = 2), DDD/1000/day (*N* = 1), and prescription counts (*N* = 1) and not per patient)
Dose	18	15	3 (DDD/1000/day (*N* = 1), TSDD^b^ (*N* = 1), medicine counts (*N* = 1))
Duration	22	22	
Indication	32	32	

^a^Defined daily dose per 1000 inhabitants per day

^b^total standardized daily dose

^*^Lassalle et al. [48]

### PPI utilization by age

**Fig. 3:**
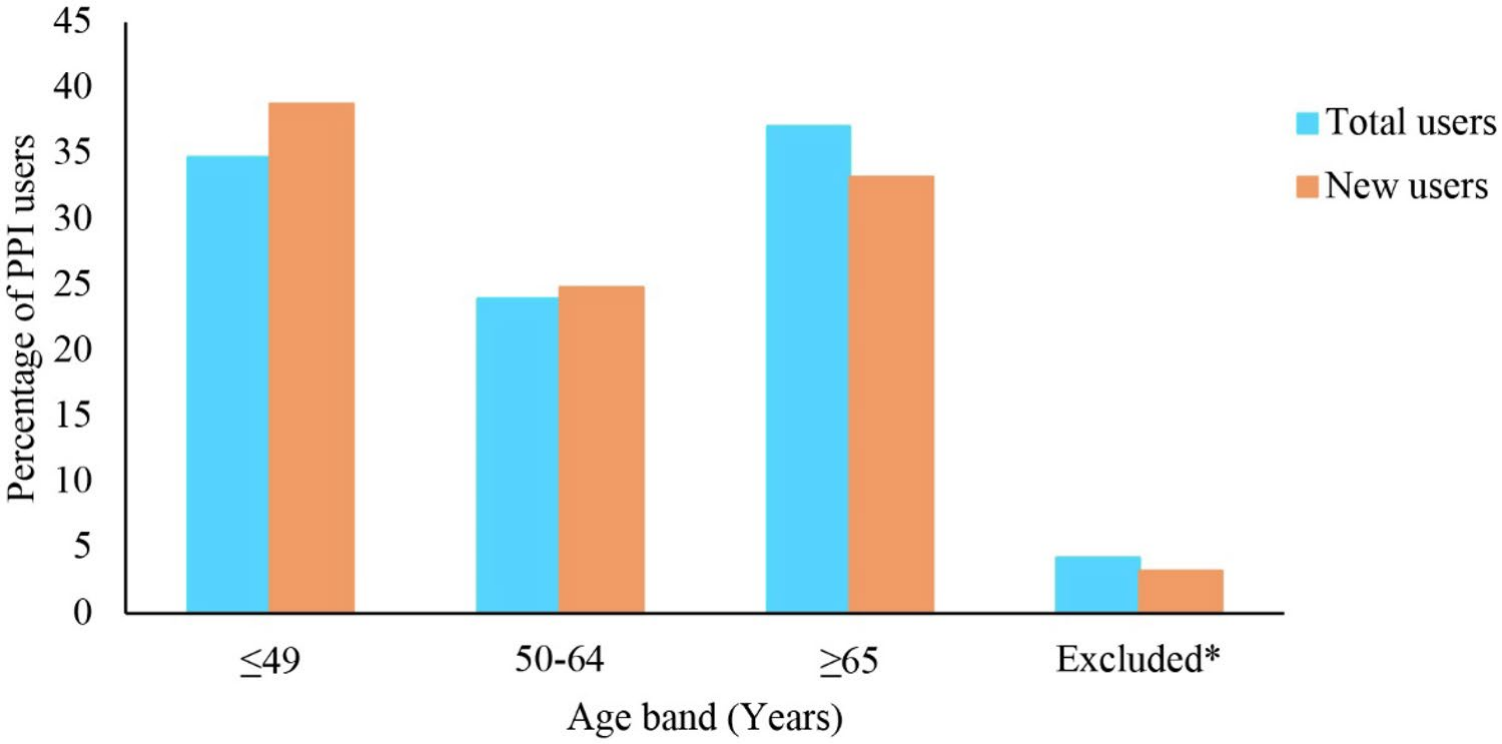
Percentage of PPI users included in this review, stratified by age groups. Total users’ (prevalent users + new users) data from 26 studies (*N* = 6,382,619); new users’ data from 15 studies (*N* = 5,060,973). *Excluded, i.e., age categories reported in eligible studies were outside the age bands used for this analysis (e.g., < 55, > 45, and 18–65) (all users = 4.2% and incident users = 3.2%)

Figure 3 depicts population utilization data across age groupings. Out of 65 studies, 26 total user (prevalent + new PPI users) studies (*N* = 6,382,619) were eligible for age analysis. Lassalle et al. were excluded from the age analysis as it highly skewed the results due to the broad binary age categorization (18–65 and > 65 years) utilized in the study [48].

Out of 6,382,619 total users, 5,060,973 (79.3%) new users (*N* = 15 studies) were included for age analysis (Fig. 3).

PPI prescriptions were most common in the oldest and young to middle age bands in the total user analysis (Fig. 3). People 65 years and older comprised 37.1% (2,367,849/6,382,619) of total users, and 34.7% of total users were ≤ 49 years (2,213,802/6,382,619). However, the percentage of young to middle aged adults (38.8%; 1,964,469/5,060,973) is higher than the older people (33.2%; 1,678,751/5,060,973) in the new user analysis (Fig. 3).

Four percent (total users; 4.2% (269,856/6,382,619) and 3.2% (new users: 162,872/5,060,973) of age data were excluded from analysis as those age data were outside the age bands used for this analysis (e.g., < 55, > 45, and 18–65) (Fig. 3).

### PPI utilization by sex

Sixty studies had sex information for both prevalent and new PPI users (*N* = 23,153,964). Among total users, 91.4% (*N* = 21,158,936) were new users (*N* = 25 studies). Over half of the population were female (total users = 56.1%; 12,990,358/23,153,964; new users = 56.1%; 11,878,481/21,158,916).

### PPI utilization by ethnicity

Nine studies provided ethnicity information for total PPI users. Seventy-five percent of the total population was White followed by Black/African American (15.6%) and Asian (1.3%). Only two studies had data for new users, and the distribution was the same as total users including that nearly 80% were White.

### PPI utilization by medication types

Thirty-one studies and 12 studies reported data for PPI types for total users and new users, respectively. Omeprazole was the leading PPI used, accounting for 44% of both total users and new users. Over one-quarter of total users (26.1%) and new users (26.6%) were prescribed esomeprazole; making it was the second-most prescribed medicine (Table 2).

**Table 2:** Summary of PPI use variables data

**Variable**	**Type of study participants**	**No. of studies**	**No. of PPI users**	***N* (%)**	
Medication types	Total users (prevalent users + new users)	31	8,461,806	Omeprazole	3,775,681 (44.6%)
				Esomeprazole	2,211,542 (26.1%)
				Pantoprazole	1,199,539 (14.2%)
				Lansoprazole	732,355 (8.7%)
				Rabeprazole	479,677 (5.7%)
				Other*	34,181 (0.4%)
				Missing	28,831 (0.3%)
	New users	12	8,237,161	Omeprazole	3,642,012 (44.2%)
				Esomeprazole	2,187,777 (26.6%)
				Pantoprazole	1,181,155 (14.3%)
				Lansoprazole	719,383 (8.7%)
				Rabeprazole	476,100 (5.8%)
				Other*	30,734 (0.4%)
				Missing	0 (0)
Dose	Total users (prevalent users + new users)	7	300,762	Higher dose	295,865 (98.4%)
Higher vs. lower dose (A)					
				Lower dose	4,865 (1.6%)
				On-demand	10 (0)
				Missing	22 (0)
Higher vs. maintenance dose (B)		3	225,280	Maintenance dose	81,805 (36.3%)
				Higher dose	143,466 (63.7%)
				Lower dose	0 (0)
				Missing	9 (0.0)
Dosing frequency (C)		7	3838	Once daily	2998 (78.1%)
				Twice daily	288 (7.5%)
				On-demand	424 (11.1%)
				As necessary	42 (1.1%)
				Other**	4 (0.1%)
				Missing	82(2.1%)
Indication	Total users (prevalent users + new users)	32	19,296,089	Gastroprotection^a^	5,527,135 (28.6%)
				Dyspepsia/GERD	1,662,931 (8.6%)
				Gastritis/duodenitis	254,351 (1.3%)
				Ulcer/GI bleeding	232,026 (1.2%)
				*H. pylori* infection	184,802 (1.0%)
				BO/ZE syndrome	9755 (0.1%)
				Uncertain/unknown indication	2,819,786 (14.6%)
				Other	101,077 (0.6%)
				Not reported	8,310,345 (43.3%)
	New users	14	18,335,098	Gastroprotection^a^	4,971,156 (27.1%)
				Dyspepsia/GERD	1,414,685 (7.7%)
				Gastritis/duodenitis	145,940 (0.8%)
				Ulcer/GI bleeding	115,664 (0.6%)
				BO/ZE syndrome	3665 (0.0%)
				Uncertain/unknown indication	2,816,788 (15.4%)
				Other	86,188 (0.5%)
				Not reported	8,466,713(46.2%)

^*^Other = dexlansoprazole or combined therapy or “Other.”

Some patients had used more than one medicine. Dose: (A) higher dose (higher and maintenance dose) = equal or greater than defined daily dose (DDD); lower dose = smaller than DDD; (B) higher dose = 40 mg/daily for omeprazole, pantoprazole, and esomeprazole; 30 mg/daily for lansoprazole; 20 mg/daily for rabeprazole (according to UK National Institute for Clinical and Care Excellence) [75]; maintenance dose = 10–20 mg/daily omeprazole, 20 mg/daily pantoprazole and esomeprazole, 15 mg/daily lansoprazole, and 10 mg/daily rabeprazole; (C) total users’ (prevalent users + new users) data from seven studies (N = 3838)

^**^Other = thrice daily/three times per week. New user analysis was not shown for dose variable. Indication Studies reported indication per patient or per PPI course. Some patients had more than one indication

^a^Gastroprotection (GI irritant medicine or treatment side effects)

*GERD* gastro-esophageal reflux disease, *GI* gastrointestinal, *BO* Barrett’s esophagus, *ZE* Zollinger–Ellison syndrome

### PPI utilization by dose

PPI dose information was available in 15 studies. Out of 15, seven (19, 24, 37, 41, 46, 56, and 79) categorized dose according to the strength of the prescribed dose, that is higher dose, maintenance dose, lower dose, and on-demand (Table 2). Moriarty et al. [56] and Cahir et al. [19] had identified pantoprazole 40 mg/daily as a higher dose while Hughes et al. [41] and Yap et al. [79] defined it as a standard dose. Because of the inconsistent reporting of doses especially higher dose, the results are shown under two analyses (i.e., higher vs. lower dose and higher vs. maintenance dose) (Table 2). Only one study provided data on new users [24]; hence, a separate new user analysis was not performed for dose variable.

Almost all users utilized the higher dose, with only approximately 2% utilizing the lower PPI dose (Table 2: dose (A)). A sub-analysis was performed using three studies (19, 24, and 56) (*N* = 225,280 of total users) that had the same definitions for higher and maintenance dosing (Table 2: dose (B)). Nearly two thirds of total users were on a higher dose (143,466/225,280; 63.7%), whereas 36.3% (81,805/225,280) were prescribed the maintenance dose. None of the studies had lower dose PPI users (Table 2: dose (B)).

Eight studies had reported dose according to the frequency of regimen (23, 25, 30, 31, 38, 42, 58, and 71). Sheikh et al. was removed from the frequency analysis as this study combined once and twice daily data [71]. Table 2: dose (C) shows that nearly eight in 10 users were prescribed a once a day regimen, followed by on-demand PPI prescriptions (11.1%).

### PPI utilization by duration

**Fig. 4:**
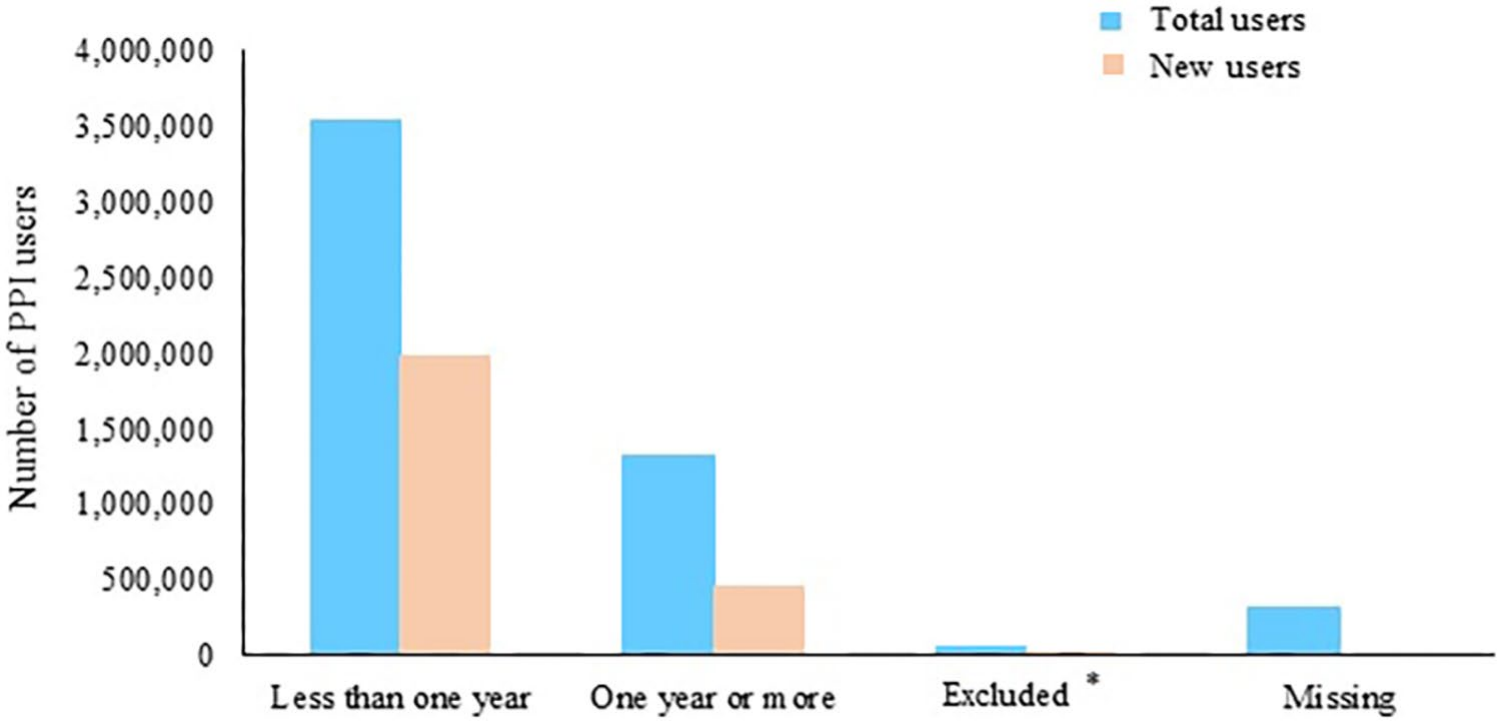
Numbers of people who used PPIs included in this review, stratified by duration groups. Total users’ (prevalent users + new users) data from 22 studies (*N* = 5,266,213); percentage of short-term users (< 1 year) vs. long-term users (≥ 1 year) (67.4% vs. 25.1%). New users’ data from 5 studies (2,450,952); percentage of short-term users (< 1 year) vs. long-term users (≥ 1 year) (80.9% vs. 18.9%; *P* < 0.001). *Excluded, i.e., duration categories reported in eligible studies were outside the duration bands used for this analysis (e.g., > 8 weeks, > 3 months, and 6–24 months) (all users = 1.3% and incident users = 0.2%)

Over 5 million total users were available for duration analysis in 22 studies. More than two-thirds were short-term users (< 1 year) (Fig. 4). Of them, nearly 45% (1,583,705/3,549,848) discontinued their PPI use within the first three months.

One-quarter of total users (1,314,544/5,266,213) were long-term users with one year or longer PPI prescriptions (Fig. 4). Among them, nearly two-thirds continued their prescription for one year to three years (65.5%; 861,357/1,314,544), while 27.8% (365,659/1,314,544) continued for more than three years.

### PPI utilization by indication

The indication for PPI prescription was described in 32 total users’ studies (Table 2). Prophylactic prescribing of PPIs for NSAIDs, antiplatelet therapy, aspirin, corticosteroid, and chemotherapy was the most prevalent indication, while dyspepsia and GERD were the second most common indication for both total and new users (Table 2). The results showed that over 2.8 million PPI users (14.6% of total users and 15.4% of new users) had uncertain or no indication for PPI prescription recorded.

Out of 65 articles, eleven studies reported the PPI utilization rates (i.e., prevalence and incidence) along with the characteristics of users that were fully generalizable to the author’s country population. The summarized results of those studies are reported in the next section.

### Prevalence of PPI utilization

Studies where prevalence rates were reported showed large variability in the percentages of population estimated to be using a PPI at a particular time point (4.4–33%) [8, 26, 33, 37, 48, 57, 60, 62, 64, 65, 72]. These were further analyzed as trends over time, with most showing a pattern of increasing use with time [8, 26, 33, 37, 48, 57, 60, 62, 64, 65, 72] (Table 3). Comparing the studies with similar observation periods showed a relatively constant annual incidence rate, while the prevalence rate continued to rise (annual incidence rate: France (Pays de la Loire region): 1.2–2.0 per 100 persons (2017–2020) [33]; Iceland: 3.3–4.1 per 100 persons (2003 to 2015) [37]; Denmark: 2.1–3.6 per 100 persons (2002–2014) [62]; Israel: 2.4–3.1 per 100 persons (2000–2015) [64]). Over the study period, the prevalence rates were two to five-fold increase in Iceland, Denmark, and Israel (Iceland: 8.5–15.5 per 100 persons [37]; Denmark: 1.8–7.4 per 100 persons [62]; Israel: 2.4–12.7 per 100 persons [64]). Similarly, PPI use increased in Spain by 44.8% from 2002 to 2015 (12.5% in 2002 to 18.1% in 2015) [72] and the USA by 18.1% from 2002/2003 to 2016/2017 (from 5.7% in 2002/2003 to 6.7% in 2016/2017) [8]. The PPI use in the UK [60] and Australia [26] were 7.7% (2014) and 12.5% (2016), respectively (Table 3).

**Table 3:** Comparison of PPI utilization variables between studies

	Author	Country	Observation period	% of population representation	Number or % of PPI users	Mean (SD)/median (IQR) age at treatment initiation	Sex (%) Female|male	Annual incidence rate Annual prevalence rate	% of long-term user
1	Daniels et al. (2020) [26]	Australia	1 July 2013–30 June 2016	100%	> 12% of the Australian population (2014 between 2016)	52 (IQR: 36–65)	60 | 40	3.9 per 100 persons (in 2016) 12.6 per 100 persons (in 2016)	25% (≥ 12 weeks) 16% (≥ 1 year)
2	Gendre et al. (2022) [33]	France	2017–2020	Represents the Pays de la Loire area	NA	67.4 (incident chronic users) 70.8 (prevalent chronic users)	54 | 46 (incident chronic users) 54 | 46 (prevalent chronic users)	1.2–2% 4.2–4.4%	NA
3	Hálfdánarson et al. (2018) [37]	Iceland	2003–2015	100%	33% (101,909/313,296)	46.0 years (IQR 30–60)	55 | 45	3.3–4.1 per 100 persons 8.5–15.5 per 100 persons	22% (≥ 1 year)
4	Rückert-Eheberg et al. (2022) [65]	Germany	2010–2018	15%	13%	NA	58 | 42 | 42.4	In 2018: 14.7% (women) 12.2% (men)	33.6% of incident users (> 6 months)
5	Lassalle et al. (2019) [48]	France	1 January 2015 between 31 December 2015	100%	29.8% (15,388,419/51,645,958)	57.0 (no SD)	57 | 43	NA	Mean treatment duration 40.9 days 4.1% (≥ 6 months)
6	Mishuk et al. (2003) [8]	USA	2002–2017	NA	6.59%	NA	58 | 41	5.70% (2002/2003)–6.73% (2016/2017)	NA
7	Muheim et al. (2021) [57]	Switzerland	1 January 2012 between 31 December 2017	~ 14%	23% of adults in 2017	51.2 (no SD)	58 | 42	19.7–23.1% (incidence of PPI prescriptions between 2012 and 2017)	NA
8	Oathman et al. (2016) [60]	UK	1 January 1990 between 31 December 2014	6%	*N* = 1,828,141	54.2 (16.3)	56 | 44	Period prevalence 0.2–15.0% Point prevalence 0.03–7.7%	26.7% (≥ 1 year) Of them, 3.9% continued for 5 years
9	Pottegard et al. (2016) [62]	Denmark	2002–2014	100%	7.4% of the adult population (2014)	NA	NA	2.15–3.64 per 100 person-years Point prevalence 1.8% to 7.4%	30% of new users (≥ 3 years)
10	Rosenberg et al. (2021) [64]	Israel	2000–2015	25%	*N* = 4,388,586	46.0 years (IQR 34–58)	56 | 44	2.4–3.1 per 100 persons 2.4–12.7 per 100 persons	15% (≥ 6 months)
11	Torres-Bondia et al. (2022) [72]	Spain	1 January 2002 between 31 December 2015		NA	62 years [21]	52 | 48	11.3–18.0% (2002–2015)	25% (> 180 DDD)

### Prescribing patterns

As seen in the full systematic review data, initiation of PPIs occurred more frequently in those of young to middle age (46 years to 57 years) [26, 37, 48, 57, 60, 62, 64, 65, 72]; however, there was an increased trend in PPI use with age particularly among adults aged 65 years and older (USA: 37.8% (≥ 65 years) vs. 2.08% (18– < 25 years) [8]; Iceland: ~ 40.0% (≥ 80 years) vs. ~ 8.0% (18–39 years) [37]; Denmark: > 20.0% (≥ 80 years) vs. ~ 1.0% (18–39 years) [62]; UK: 23.0% (≥ 80 years) vs. 1.0% [18–30] [60]; Australia: 42.8% (≥ 85 years) vs. 4.5% (18–34 years) [26]; Germany: ~ 40.0% (≥ 90 years) vs. < 5% (20– < 25 years) [65]; Spain: 61.0% (≥ 65 years) vs. 2.0% (16–24 years)) [72]. The prevalence of PPI use is more common among females [26, 37, 48, 57, 60, 62, 64, 65] (Table 3).

While omeprazole remained the most used PPI in almost all countries, Denmark [62], Switzerland [57], and Germany (Bavaria region) [65] had a greater utilization of pantoprazole. Over the time periods, some shift from omeprazole (racemate) to esomeprazole (isomer) was seen (Table 3).

## Doses/duration

Most PPI users maintained their initial strength throughout the course of treatment without any dose adjustments [26, 37]. It was found that a larger proportion of higher dose users remained on this dose for a longer period. In Iceland, 13.0% of higher dose users continued the same dose for five years, in contrast to 2.0% of lower dose users [37].

Most of the studied countries had a considerably large proportion of long-term users (16.0%, 22.0%, and 26.7% of users continued for at least one year in Australia, Iceland, and the UK, respectively) [26, 37, 60] (Table 3).

Long-term use of PPIs increased with age (e.g., in Iceland, 36.0% of people older than 80 years continued the treatment for one year after initiating the PPI prescription, compared to 13.0% aged 19–39 years) [37]. The same kind of age pattern was observed in Denmark [62], Israel [64], France [48], and Spain [72].

## Discussion

This systematic review is the first to describe global PPI use patterns by demographics and medication factors and is based on published literature over three decades. The findings of this review provide robust evidence on actual PPI use in the general population, which in turn provides information to develop and update PPI prescribing policies to improve the safety of PPI use.

In this review, 65 papers were analyzed and generated a total PPI user group of 28 million across 23 countries, representing 23.4% of the adult population. The results showed that the prevalence of PPI prescribing has steadily increased from 1990; however, the rates have declined recently in some countries, e.g., Germany [65], the USA [8], and Spain [72]. The incidence rate of PPI prescribing remained relatively stable over time, implying that the observed higher prevalence rate is due to a growing number of long-term users. The observed rate stabilization could be attributed to the U.S. Food and Drug Administration’s [80, 81] safety warnings and recent findings from observational studies highlighting the potentially deleterious consequences of long-term usage [80, 81].

PPI use was largest among adults aged 65 years and older, followed by the young to middle aged group (≥ 65 years old: 37.1% of total users and ≤ 49 years old: 34.7% of total users, respectively) (Fig. 3), females (56.1% of total users), and White ethnicity (75.0% of total users).

Despite clinical guidelines recommending the lowest possible dose for the shortest duration (generally 4–8 weeks) [2, 8], this systematic review has observed PPIs being prescribed in higher doses (63.7% of total users) and for longer periods (≥ 1 year: 25.1% of total users). Of long-term users, 65.5% continued for up to 3 years, and 27.8% continued for over three years. These findings were consistent with the national level data and showed that 16.0–26.7% of PPI users had continued therapy for longer than one year [26, 37, 60],

The data of this review is insufficient to find the specific reason for the use of PPIs at an individual patient level. Increased prevalence of GERD in younger patients [82] and inappropriate prescribing for stress ulcer prophylaxis [48, 83] or co-prescribing with NSAIDs [48] or no indication [65] may increase PPI use, particularly in young and middle-aged groups.

The global prevalence of PPI use is increased among older adults and may be as a result of multiple comorbidities, increased risk of acid-related gastrointestinal disorders, polypharmacy, and lack of deprescribing [84, 85]. Recent studies show both the need and emphasis on reducing inappropriate use of PPIs in those over 65. Hence, deprescribing of inappropriate PPIs in older adults is a growing area of interest [86, 87].

It is important to note that much of the data for this analysis came from “Western” countries (North America, Europe, and Oceania) and data from Africa, Latin America, Russia, and some parts of Asia (China, India, and Pakistan) were not available. Market research reports indicate the Asia Pacific region holds the fastest growing PPI market [88, 89], while Latin America and the Middle East regions are more likely to have a large PPI market due to increased GERD prevalence [90]. Hence, a greater understanding of PPI use in these countries is warranted.

Esomeprazole—(S)-isomer of omeprazole—has an identical mechanism of action and is a therapeutically interchangeable high-cost medicine. However, this was the second most common PPI found in this review. When there is no clinical benefit to an alternate medicine, prescribers should consider cost and patients’ affordability when prescribing medicine.

Limited data on PPI user’s ethnicity variable limits the opportunity to calculate PPI use rates per ethnicity. Ethnicity was recorded for 215,119 users (out of 78,151,104 of the source population), representing only 0.3% of the source population. Hence, more studies are warranted to investigate the PPI use by ethnicity.

Another scope of the analysis was to evaluate the dose and duration of PPI use by indication, which would explicitly provide information on whether the prescribing has adhered to the current treatment guidelines [2, 3, 9]. However, this sub-analysis was not feasible as none of the studies provided data for this hypothesis. Hence, further work is required to understand the reasons for PPI use at a higher dose and longer than the guidelines recommend.

The current review provided evidence for only prescribed PPIs but not for OTC (i.e., available via a pharmacy/supermarket without prescription) use. The prescription use of PPIs dates back to 1988; however, OTC availability was initiated in the early twenty-first century and is seen in many countries, including the USA, the UK, Sweden, and New Zealand [91–93]. Generally, OTC medicines are more expensive, and a supply period is less than a month. As this review assessed the long-term use of PPI, OTC use was not considered. However, further studies are required to understand the safety and the magnitude of global OTC use of PPIs.

### Clinical implications

Based on the findings, this review recommends three key clinical practices for healthcare professionals to mitigate the overuse of PPIs. First, regular reviewing of PPI prescription and documenting indications for continual PPI use. This helps recognize whether the patient still has the initial indication for prescribing, or if the medicine was intended for a short-term use only.

Second, deprescribing (stopping or stepping down or reducing to intermittent use, on-demand use, or lower dose) of PPIs, when there is no appropriate ongoing indication or evidence of benefit [3, 94]. The NICE guidelines recommend a minimum yearly review of PPI prescriptions, and any unindicated drug should be stopped or stepped down if possible [3]. This decreases the treatment cost, unnecessary health harms, and also prevent clinically significant drug interactions including situations where concomitant use of omeprazole will increase the level (e.g., mavacamten) or decrease the effect (e.g., clopidogrel) of medicines by affecting CYP2C19 enzyme metabolism and by increasing gastric pH (e.g., increase the effect of digoxin) [95].

The risk of rebound acid hypersecretion after abrupt withdrawal of long-term therapy [9] discourages patients from stopping the treatment. Dills et al., in their systematic review, found that PPIs are one of the drugs most resistant to deprescribing [96]. Hence, educating and empowering patients about the reasons for deprescribing ae important to ensure the success of the action.

### Strengths and limitations

This systematic review is the first to describe the global PPI use in the general population of more than 28 million users from 65 studies in 23 different countries. While several studies have reported on the irrational use of PPIs, the increased use of PPIs, and the increased spending on PPIs [18, 26, 37, 48, 60, 62, 97], no other study has evaluated the global utilization of PPIs (including user demographics, magnitude of use, and clinical use patterns) within the general population.

The limitations should be acknowledged. First, we could not investigate the PPI prescribing disparities across income, education level, other comorbidities, and body mass index (BMI). These variables were not routinely reported in the studies analyzed. Second, there was data loss when finding the best fitting age bands, which was necessary due to the inconsistent age groups used within different studies. Attempts were made to align age bands for this analysis such that the least amount of data (total users = 4.2%, new users = 3.2%) was lost while still providing sensible age groupings.

Third, while the national and regional studies with generalizable populations compared in this review provide insight into country specific trends, it is important to acknowledge differences in observation periods. Additionally, it is not possible to determine policy changes, funding, and availability status which may have influenced the prescribing of PPIs.

## Conclusion

Global PPI use is significant, with nearly 25% of the adult source population prescribed a PPI. PPI use was reported across the adult life span, with 63% of users being under the age of 65 and 37% being over the age of 65. Females and those of “White” ethnicities had the highest use; however, this may be influenced by the populations studied in the published literature. Most users were on higher doses, and 25% of PPI users continued therapy for longer than one year, with almost a third of patients continuing over three years. Omeprazole was the most frequently prescribed PPI, followed by esomeprazole.

Given the widespread use of this medication and increasing concern regarding long-term use, the review provides a catalyst to support more rational use, particularly with unnecessarily prolonged use.

## Supplementary Information

Below is the link to the electronic supplementary material.

Supplementary file1 (DOCX 45 KB)

Supplementary file2 (DOCX 18 KB)

## Data Availability

The datasets during and/or analyzed during the current study are available from the corresponding author on reasonable request.
