# Supplementary material for: Proton pump inhibitor use: systematic review of global trends and practices
Source: Eur J Clin Pharmacol. 2023 Jul 7;79(9):1159–72. doi: 10.1007/s00228-023-03534-z (PMC10427555; doi:10.1007/s00228-023-03534-z)
Supplement: Supplementary file 1 — Supplementary file1 (DOCX 45 KB) [file 228_2023_3534_MOESM1_ESM.docx]

Supplementary Table 1: Number of studies eligible for the analysis

| **No** | **Study (First author/**  **Publication year)** | **Country** | **Continent** | **Study period** | **Study design** | **Study setting** | **Total number of PPI users (Final analysis)** | **PPI utilisation variable** |
| --- | --- | --- | --- | --- | --- | --- | --- | --- |
| 1 | Antoniou TP, 2015 | Ontario, Canada | North America | April 2002 to November 2011 | Population-based cohort study | Ontario residents | 290,592 | Age, Sex, Indication  PPI types |
| 2 | Biyik M, 2017 | Turkey | Asia | October 2011 to September 2012 | Case–control study | Outpatient clinic | 154 | Sex, Indication, PPI types |
| 3 | Brusselaers N, 2018 | Sweden | Europe | July 2005 to December 2014 | Population-based cohort study | General population  in Sweden | 796,492 | Age, Sex, Indication, Duration |
| 4 | Cahir C, 2012 | Republic of Ireland | Europe | 2007 | Retrospective population-based cohort study | General population  in Ireland | 167,747 | Age, Dose, Duration |
| 5 | Casula M, 2018 | Italy | Europe | January 2003 to December 2007; follow-up till December 2010 | Nested case-control | Resident  in Lombardy | 106,992 | Sex, PPI types |
| 6 | Chan A, 2018 | British Columbia, Canada | North America | April 2015 to March 2016 | Retrospective cross-sectional study | Residential care sites | 331  (PPI orders = 407) | Sex, Indication |
| 7 | Chen Y, 2016 | NSW, Australia | Oceania | January 2006 to June 2012 | Prospective cohort study | Participants from Sax Institute’s 45 and Up Study | 23,257 | Age, Sex, PPI types |
| 8 | Chey WD, 2009 | Massachusetts, USA | North America | July 2007 to August 2007 | Survey | Participants from four different community health plans | 617 | Sex, Indication, Dose, Ethnicity, PPI types |
| 9 | Claessens AAMC, 2000 | Netherlands | Europe | January 1994 to April 1998 | Prospective observational study | Lansoprazole first users in Netherlands | 10,008 | Age, Sex, Indication, Dose, PPI types |
| 10 | Clooney AG, 2016 | Manitoba, Canada | North America | Not mention | Population-based database | Manitoba Health population registry | 32 | Sex, Indication, Dose, PPI types |
| 11 | Daniels B, 2020 | Australia | Oceania | July 2013 to June 2016 | Population-based cohort study | General population  in Australia | 4,388,586 | Age, Sex, Dose, Duration |
| 12 | Davies M, 2008 | England | Europe | September 2000 to April 2001 | Post marketing surveillance study | Esomeprazole first users in England | 11, 595 | Sex, Indication, PPI types |
| 13 | de Vries F, 2009 | UK | Europe | January 1988 to February 2007 | Retrospective cohort study | General practice Research Database in UK | 234,144 | Sex, PPI types |
| 14 | Ding J, 2014 | Pennsylvania, USA | North America | January 1999 to December 1999 | Retrospective cohort study | Participants from PACE program, Pennsylvania state | 1,604 | Age, Sex, Ethnicity |
| 15 | Doell A, 2018 | Manitoba, Canada | North America | October 2016 | Cross-sectional study | Long-term care homes | 147 | Sex, Indication, Dose, PPI types, Duration |
| 16 | Fattahi MR, 2019 | South Iran | Asia | June 2015 to March 2019 | Analytical cross-sectional study | Gastroenterology clinic | 133 | Age, Sex, Dose, Duration |
| 17 | Gadzhanova SV, 2009 | Australia | Oceania | July 2004 to June 2007 | Retrospective cohort study | Pharmacy claims data of veterans | 41,041 | Age, Sex, Duration |
| 18 | Gendre P, 2022 | France | Europe | 01 October 2016 and 31 December 2020 | Cohort study | General population living in the Pays de la Loire area | 77,222 | Sex |
| 19 | Gomm W, 2016 | German | Europe | 2004-2011 | Prospective cohort study | Largest Health insurance database in German | 2950 | Sex, PPI types |
| 20 | Gray SL, 2016 | Northwest, USA | North America | 1994 to 2004; follow up till 2014 | Prospective population-based cohort study | Participants from ACT study | 1061 | Sex, Dose, Ethnicity, PPI types, Duration |
| 21 | Haenisch B, 2015 | German | Europe | 2003 | Longitudinal multicentre cohort study | General practitioners’ registries at six sites | 713 | Sex |
| 22 | Halfdanarson OO, 2018 | Iceland | Europe | January 2003 to December 2015 | Nationwide population-based drug-utilization study | Outpatient care dispensing data in Iceland | 101,909 | Age, Sex, Dose, PPI types, Duration |
| 23 | Hendrix I, 2019 | South Australia | Oceania | April 2014 to August 2014 | Secondary analysis of Cross-sectional study | Six residential aged care services | 196 | Sex, Indication, Dose, PPI types, Duration |
| 24 | Hermos JA, 2012 | New England, USA | North America | October 1996 to September 2007 | Retrospective, nested case-control design | Pharmacy and administrative database | 16,984 | Age, Sex, Indication, Duration |
| 25 | Hong KS, 2013 | Seoul, South Korea | Asia | January 2005 to December 2009 | Part of a hospital-based longitudinal cohort study | Tertiary care  centre | 61, 834 | Sex, Indication, Duration |
| 26 | Hughes JD, 2009 | Western Australia | Oceania | April 2005 to June 2005 | Observational study | Teaching hospital | 97 | Sex, Indication, Dose, PPI types |
| 27 | Jarbol DE, 2019 | Southern Denmark | Europe | Autumn 2016 | Prospective audit | General practitioners in the Southern Denmark | 1842 | Age, Sex, Indication, Dose, PPI types, Duration |
| 28 | Jena AB, 2012 | USA | North America | 1997 to 2007 | Retrospective claims-based cohort study | Six private U.S. health plans | 26,436 | Sex |
| 29 | Kim S, 2015 | South Korea | Asia | January 2007 to June 2012 | Retrospective study | Teaching hospital | 112 (105) | Age, Sex, Indication, PPI types, Duration |
| 30 | Koggel LM,2022 | Netherlands | Europe | 2016-2018 | Cohort study | Primary care  in Leiden/The Hague region | 23,601 | Age, Sex, Indication, Duration |
| 31 | Kojima Y, 2018 | Japan | Asia | February 2015 to June 2016 | A multicentre Prospective cross-sectional study | General hospital | 212 | Sex, Dose, Duration |
| 32 | Larsen MD, 2014 | Denmark | Europe | January 2009 to December 2010 | Register study | Tertiary teaching hospital | 5387 | Sex, PPI types |
| 33 | Lassalle M, 2020 | France | Europe | January 2015 to December 2015 | Nationwide drug utilization study | General population  In France | 15,388,419 | Age, Sex, Indication, PPI types |
| 34 | Lee J, 2016 | USA | North America | 2001 and 2008 | Cross-sectional study | Intensive care unit | 3,725 | Sex, Ethnicity |
| 35 | Ma J, 2008 | Canada | North America | January 2004 to December 2004 | Observational cohort study | Canadian Forces database | 4738 | Sex, Duration |
| 36 | Machado-Alba J, 2013 | Colombia | South America | October 1, 2010, to October 31, 2010 | Cross-sectional study | Colombian Health System | 113560 | Sex, PPI types |
| 37 | Mafi JN, 2019 | USA | North America | 2018 | Cross-sectional study | Primary care | 8729 (Final N=399) | Sex, Indication, Ethnicity |
| 38 | Mares-Garcia E, 2017 | Alicante, Southeast of Spain | Europe | August to October 2013 | Cross-sectional observational study | Three community pharmacies | 302 | Sex |
| 39 | Martin RM, 2000 | England | Europe | June 1989 to June 1990 (Ome)  May to November 1994 (Lanso) December 1996 to June 1997 (Panto) | Prescription-event monitoring cohort studies | First PPI users in England | 45,075  Omeprazole=16,205  Lansoprazole=17,329Pantoprazole=11,541 | Sex, Indication, PPI types |
| 40 | Martin RM, 1998 | England, Wales, Scotland | Europe | October 1991 to September 1996 | Secondary analysis of cross-sectional survey | General practitioners’ data | 8811 | Age, Sex, Indication |
| 41 | Mishuk AU, 2021 | USA | North America | 2002-2017 | Cross-sectional study | Medical Expenditure Panel Survey (MEPS) data | 316,841,496 | Age, Sex, Ethnicity |
| 42 | Moriarty F, 2016 | Ireland | Europe | 1997-2012 | Repeated cross-sectional study | Administrative pharmacy claims data | (2012) 64,842 | Indication, Dose, Duration |
| 43 | Muheim L, 2021 | Switzerland | Europe | 1 January 2012 to 31 December 2017 | Population-based cohort study | Swiss general population | 671,683 | Sex |
| 44 | Nguyen PVQ, 2018 | Quebec, Canada | North America | June 2016 to March 2017 | Prospective cross-sectional study | Emergency departments | 871 | Sex, Indication, Dose, PPI types |
| 45 | Nishtala PS, 2015 | New Zealand | Oceania | January 2005 to December 2013 | Repeated cross-sectional study | General population in New Zealand | (2013) 211 031 | Age, Sex, Ethnicity, Dose, PPI types |
| 46 | Othman F, 2016 | UK | Europe | January 1990 to December 2014 | Repeated cross-sectional study | Data from primary care practices in UK | 1,828,141 | Age, Sex, Indication, Duration |
| 47 | Pasina L, 2016 | Italy | Europe | January to May 2015 | Cross-sectional study | Nine community pharmacies | 260 | Sex, Indication, PPI types, Duration |
| 48 | Pottegård A, 2016 | Denmark | Europe | January 2002 to December 2014 | Descriptive utilization study | General population in Denmark | 1,617,614 | Age, Sex, PPI types, Duration |
| 49 | Poulsen AH, 2009 | North Jutland, Denmark | Europe | 1990 to 2003 | Population-based cohort study | General population in North Jutland County | 18,790 | Age, Sex, PPI types |
| 50 | Rosenberg V, 2021 | Israel | Asia | 1 January 2000 to 31 December 2015 | Population-based cohort study | General population in Israel | 528,420 | Age, Sex, Indication |
| 51 | Rückert-Eheberg IM 2022 | Germany | Europe | 2010-2018 | Cross-sectional study | Statutory health insurance (SHI) | 571,924 | Age, Sex, Indication |
| 52 | Sanchez-Cuen JA, 2013 | Mexico | North America | February to May 2012 | Prospective cross-sectional study | Outpatient care | 153 | Sex, Indication, PPI types |
| 53 | Sarzynski E, 2011 | USA | North America | January 2004 to December 2006 | Retrospective cohort study | Outpatient care | 98 | Sex, Indication, PPI types |
| 54 | Schneider JL, 2015 | USA | North America | (Pantoprazole) January 2000 to December 2003 (Other PPI) January 1996 to December 2003 | Cohort study | In-patient and out-patient services | 61,864  Pantoprazole=34,178  Other PPI=27,686 | Age, Sex, PPI types |
| 55 | Seo SI, 2021 | Korea | Asia | 2002-2013 | Population-based cohort study | National Health Insurance Service database | 101,438 | Age, Sex |
| 56 | Shah NH, 2015 | USA | North America | STRIDE=1994 to 2011  PF=2007 to 2012 | Data mining study | University hospital | STRIDE=32,363  PE=74,516 | Age, Sex, Ethnicity, PPI types |
| 57 | Sheikh I, 2014 | Ohio, USA | North America | 2011 (4 months) | Survey | Multispecialty clinics | 610 (Final N= 413) | Age, Sex, Dose, Ethnicity |
| 58 | Torres-Bondia, F, 2020 | Spain | Europe | 1st January 2002 to 31st December 2015 | Retrospective cohort study | General population in Lleida County | 215,417 | Age, Sex, PPI types |
| 59 | Van Boxel OS, 2009 | Netherlands | Europe | January 2006 to June 2007 | Retrospective cohort study | Health Insurer in Netherlands | 236,122 | Age, Sex |
| 60 | Van Soest EM, 2006 | Netherlands | Europe | January 1996 to December 2003 | Retrospective cohort study | General practitioners’ data | 16,311 (Final N=10,833) | Sex, Indication, Duration |
| 61 | Wallerstedt SM, 2017 | Västra Götaland, Sweden | Europe | July 2005 to December 2010 | A cross-sectional population-based study | Four population level databases | 32,421 | Sex, Indication, PPI types |
| 62 | Wang YT et al. | Taiwan | Asia | January 1999 to December 2013 | Nationwide population-based retrospective cohort study | National Health Insurer in Taiwan | 52,389 | Age, Sex, PPI types |
| 63 | Wei J, 2020 | UK | Europe | January 2000 to December 2016 | A population-based cohort study | General practitioners’ data in UK | 50,265 | Sex |
| 64 | Xie Y, 2016 | USA | North America | October 2006 to September 2008 | Cohort study | Department of Veterans Affairs (VA) national databases | 173,321 | Sex, Indication, Ethnicity, PPI types |
| 65 | Yap MH, 2019 | Melbourne, Australia | Oceania | June to July 2016 | A single centre prospective observational study | Tertiary hospital | 198 | Sex, Indication, Dose, PPI types, Duration |
