# Supplementary material for: Proton pump inhibitor use: systematic review of global trends and practices
Source: Eur J Clin Pharmacol. 2023 Jul 7;79(9):1159–72. doi: 10.1007/s00228-023-03534-z (PMC10427555; doi:10.1007/s00228-023-03534-z)
Supplement: Supplementary file 2 — Supplementary file2 (DOCX 18 KB) [file 228_2023_3534_MOESM2_ESM.docx]

EMBASE AND MEDLINE AND INTERNATIONAL PHARMACEUTICAL ABSTRACT (IPA)

(proton-pump inhibitor or proton pump inhibitor or proton-pump inhibitors or proton pump inhibitors or omeprazole or pantoprazole or lansoprazole or esomeprazole or PPI or PPIs or acid-suppressant or acid-suppressing or acid suppressant or acid suppressing)

and

(dose or duration or frequency or indication or utilisation or utilization or adherence or compliance)

and

(ecological or audit or survey or observational or cross sectional or cross-sectional or cross-sequential or cross sequential or case-control or case control or cohort or longitudinal study or longitudinal-study or systematic review or health data or dispensing data or prescription data or claims data)

and

(long term or long-term or community or general practice or general practitioner or GP or admission or primary care or population-based or population based or nationwide or primary health or population-level or region)

and

**Afghanistan** or **Albania** or **Algeria** or **Andorra** or **Angola** or **Antigua or Barbuda** or [**Argentina**](https://theworldtravelguy.com/category/destinations/south-america/argentina/) or **Armenia** or [**Australia**](https://theworldtravelguy.com/category/destinations/oceania/australia/) or **Austria or Azerbaijan** or **Bahamas** or **Bahrain** or **Bangladesh** or **Barbados** or **Belarus** or **Belgium** or [**Belize**](https://theworldtravelguy.com/category/destinations/central-america/belize/) or **Benin or Bhutan or Bolivia** or **Bosnia or Herzegovina** or **Botswana** or [**Brazil**](https://theworldtravelguy.com/iguazu-falls-argentina-brazil/) or [**Brunei**](https://theworldtravelguy.com/destinations/brunei-travel-guide/) or **Bulgaria** or **Burkina Faso** or **Burundi or Cabo Verde** or [**Cambodia**](https://theworldtravelguy.com/destinations/cambodia-travel-guide/) or **Cameroon** or **Canada** or **Central African Republic** or **Chad** or **Chile** or **China** or **Colombia** or **Comoros or Congo** or **Costa Rica or Cote Ivoire or Croatia** or **Cuba** or **Cyprus** or **Czech Republic** or **Denmark** or **Djibouti** or **Dominica** or **Dominican Republic or East Timor or Ecuador or** [**Egypt**](https://theworldtravelguy.com/destinations/egypt-travel-guide-the-best-egypt-travel-tips-blog/) or **El Salvador** or **Equatorial Guinea** or **Eritrea or Estonia** or **Eswatini** or **Ethiopia** or **Fiji** or **Finland** or **France** or **Gabon** or **Gambia** or **Georgia** or **Germany or Ghana or** [**Greece**](https://theworldtravelguy.com/category/destinations/europe/greece/) **or Grenada** or [**Guatemala**](https://theworldtravelguy.com/category/destinations/central-america/guatemala/) or **Guinea** or **Guinea-Bissau** or **Guyana or Haiti or Honduras** or **Hungary** or [**Iceland**](https://theworldtravelguy.com/destinations/iceland-travel-guide-travel-tips-blog-for-traveling-to-iceland/) or [**India**](https://theworldtravelguy.com/destinations/india-travel-tips-complete-india-travel-guide/) or [**Indonesia**](https://theworldtravelguy.com/destinations/indonesia-travel-guide/) or **Iran** or **Iraq** or **Ireland** or **Israel** or **Italy or Jamaica** or [**Japan**](https://theworldtravelguy.com/best-things-to-do-in-japan-what-to-do-for-fun/) or [**Jordan**](https://theworldtravelguy.com/destinations/jordan-travel-guide/) or **Kazakhstan** or **Kenya** or **Kiribati or Korea or Kosovo** or **Kuwait or Kyrgyzstan or Laos or Latvia** or [**Lebanon**](https://theworldtravelguy.com/destinations/lebanon-travel-guide/) or **Lesotho** or **Liberia** or **Libya** or **Liechtenstein or Lithuania or Luxembourg** or **Madagascar** or **Malawi** or [**Malaysia**](https://theworldtravelguy.com/destinations/malaysia-travel-guide/) or **Maldives or Mali** or **Malta** or **Marshall Islands** or **Mauritania** or **Mauritius or Mexico or Micronesia** or **Moldova** or **Monaco** or **Mongolia** or **Montenegro** or **Morocco** or **Mozambique** or [**Myanmar**](https://theworldtravelguy.com/destinations/myanmar-travel-guide-best-tours-and-travel-tips/) **or Burma** or **Namibia** or **Nauru** or [**Nepal**](https://theworldtravelguy.com/destinations/nepal-travel-guide/) **or** [**Netherlands**](https://theworldtravelguy.com/castle-de-haar-kasteel/) or [**New Zealand**](https://theworldtravelguy.com/destinations/new-zealand-travel-guide-best-travel-tips-blog/) or **Nicaragua** or **Niger** or **Nigeria** or **North Macedonia or Norway or Oman or Pakistan or Palau** or **Panama** or **Papua New Guinea** or [**Paraguay**](https://theworldtravelguy.com/saltos-del-monday-falls-paraguay/) or [**Peru**](https://theworldtravelguy.com/category/destinations/south-america/peru/) or [**Philippines**](https://theworldtravelguy.com/destinations/philippines-travel-guide-the-best-philippines-travel-tips-blog/) or **Poland** or **Portugal** or [**Qatar**](https://theworldtravelguy.com/destinations/qatar-travel-guide/) or **Romania** or **Russia or Rwanda** or **Saint Kitts or Saint Lucia or Saint Vincent or Grenadines or Samoa** or **San Marino** or **Sao Tome or Saudi Arabia** or **Senegal** or **Serbia or Seychelles or Sierra Leone or** [**Singapore**](https://theworldtravelguy.com/things-to-do-in-singapore-airport/) or **Slovakia** or **Slovenia** or **Solomon Islands** or **Somalia** **South Africa** or [**Spain**](https://theworldtravelguy.com/destinations/spain-travel-guide-spain-travel-tips-blog-info/) or **Sri Lanka or Sudan or Suriname** or **Sweden** or **Switzerland** or **Syria** or Taiwan or **Tajikistan** or **Tanzania** or [**Thailand**](https://theworldtravelguy.com/destinations/thailand-travel-guide-the-best-thailand-travel-tips/) **or Togo or Tonga** or **Trinidad or Tobago** or **Tunisia or** [**Turkey**](https://theworldtravelguy.com/destinations/turkey-travel-guide/) or **Turkmenistan** or **Tuvalu or Uganda** or **Ukraine** or [United Arab Emirates](https://theworldtravelguy.com/destinations/uae-dubai-travel-guide/) or **United Kingdom or** [United States of America](https://theworldtravelguy.com/category/destinations/north-america/united-states/) or [Uruguay](https://theworldtravelguy.com/colonia-del-sacramento-uruguay/) or **Uzbekistan** or **Vanuatu** or Vatican City or **Venezuela** or [Vietnam](https://theworldtravelguy.com/destinations/vietnam-travel-guide-the-best-vietnam-travel-tips-blog/) or **Yemen or Zambia or Zimbabwe**
